# The 2021 Performance Report of the *Malaysian Journal of Medical Sciences*

**DOI:** 10.21315/mjms2022.29.4.1

**Published:** 2022-08-29

**Authors:** Zulkapli Nour Azimah, Jafri Malin Abdullah

**Affiliations:** 1 Malaysian Journal of Medical Sciences, Universiti Sains Malaysia, Pulau Pinang, Malaysia; 2 Malaysian Journal of Medical Sciences, Universiti Sains Malaysia, Kelantan, Malaysia

**Keywords:** journal performance, submission trend, journal impact

## Abstract

This is a report on the annual performance of the *Malaysian Journal of Medical Sciences* (MJMS) in 2021. It includes the important metrics of journal impact measurement and the appointment of experts within their respective fields as new members of the Editorial Board.

## Introduction

The Editorial Office presents this report on the performance of the *Malaysian Journal of Medical Sciences* (MJMS) throughout 2021. Data were extracted from the manuscript submission platform and the calculation data of research metrics by various companies were searched through the Internet.

### Submission Trend of New Manuscripts

**Figure 1:**
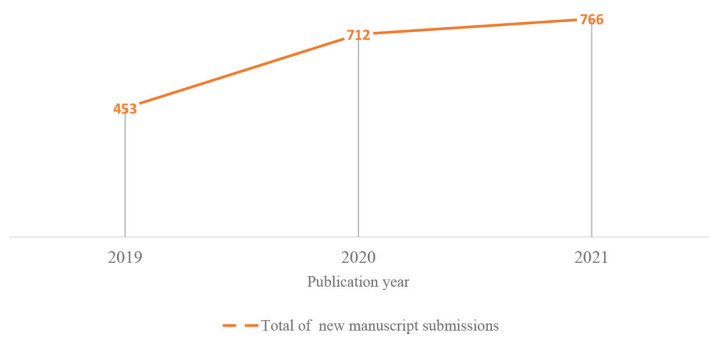
Annual total submissions of new manuscripts for 2019–2021 Source: https://mc.manuscriptcentral.com/maljms

In the publication year of 2021, MJMS received a total submission of 766 new manuscripts, which was 7% higher than in the preceding year (1, 2). However, this may be considered a slight increment compared to the total submission in 2020, which was 36.4% higher than that in 2019 (Figure 1).

#### Submission Pattern by Manuscript Type

**Figure 2:**
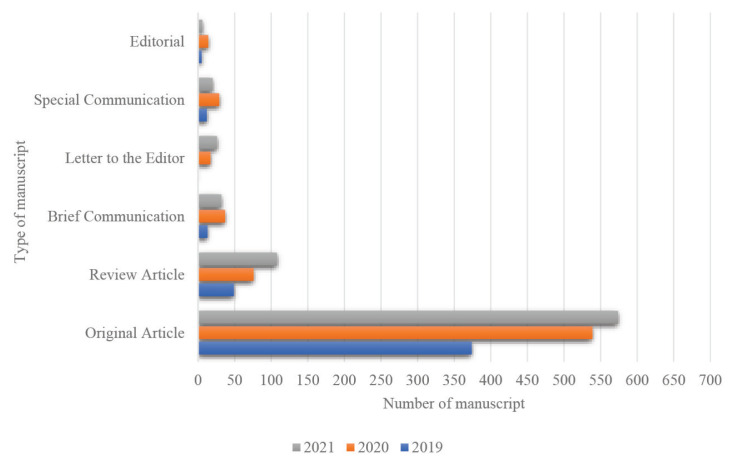
Submission trends by manuscript type in 2019–2021 Source: https://mc.manuscriptcentral.com/maljms

Original Article manuscripts comprise the majority of manuscript types submitted to MJMS for the past years, followed by Review Article manuscripts. In particular, the submission of Original Article manuscripts has increased by 30.6% in 2020 compared to submissions in 2019 and showed a slight increase of 6% in 2021. The same submission trend is observed for Review Articles, as shown in Figure 2.

**Figure 3:**
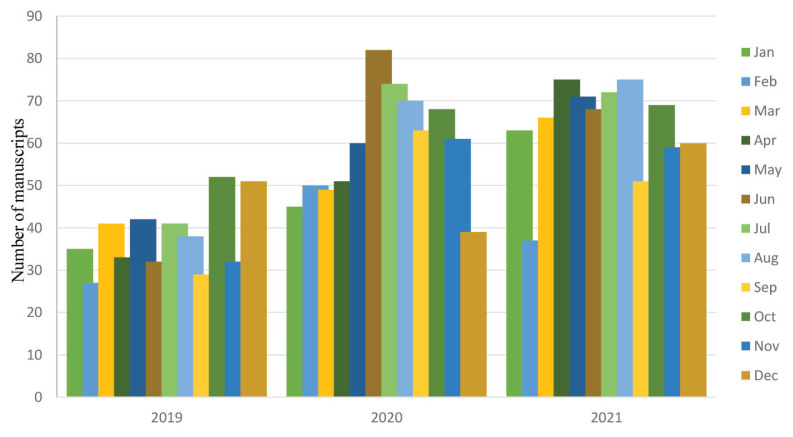
Comparison of the patterns of new manuscript submissions for 2019–2021 Source: https://mc.manuscriptcentral.com/maljms

The average number of manuscript submissions in 2021 was 64, in which the same highest number of submissions (75 manuscripts) were recorded in April and August, respectively, compared to other months. A comparison of submission patterns of the preceding years is shown in Figure 3.

#### Contributions by Country

Manuscript contributions to MJMS are exclusively led by researchers from Malaysia. Table 1 shows the contributions of researchers from other countries and world regions apart from Malaysia in the most recent 3-year period (3). Despite the huge number of manuscripts respectively submitted by each country, the percentages of rejection are also considered high (Table 2).

#### Contributions by Organisation

Universiti Sains Malaysia is the local institution that has contributed the highest number of manuscripts to MJMS ever since it started publication. Other local institutions that can be considered major manuscript contributors to MJMS are Universiti Kebangsaan Malaysia and the Ministry of Health Malaysia, followed by foreign institutions, such as the University of Indonesia and Islamic Azad University of Iran, as shown in Table 3 (3).

### Research Metrics

According to the Academic Accelerator (4), the 2021–2022 Impact IF of MJMS is 1.387. This score increased by 34.20% compared to 0.913 in 2019–2020. The best quartiles of 2021–2022 are Q2 and Q3 for the subcategories Medicine (all) and Medicine (miscellaneous), respectively.

The H-index is a means of measuring the productivity and citation impact of a publication. According to the SCImago Journal Rank (SJR) (5), the H-indexes of MJMS in the years 2020 and 2021 are 25 and 30, respectively. This means that 30 articles in this journal have more than 30 citations.

The SJR is an indicator that measures a journal’s scientific influence. It considers the number of citations received by a journal and the importance of the journals from which these citations come. According to SJR, MJMS has a ranking 0.433 in 2021 compared to 0.394 in 2020.

The impact score of an academic journal, also denoted as the journal impact score (JIS), is a measure of the annual average number of citations in recent articles published in that journal. The impact score of MJMS in 2021 is 1.82. This impact score, which is based on Scopus data, increased by a factor of 0.43. Furthermore, the approximate percentage change is 30.94% compared to the preceding year (i.e. 2020), indicating a rising trend (Table 4) (6).

CiteScore is a metric for measuring journal impact in Scopus. It is measured within a 4-year citation window and is calculated from the Scopus journal list, which covers all subject areas. Table 4 shows the value trends of CiteScore in 2019–2021 (6).

### New Members of the Editorial Board

MJMS welcomes Dr. Evelyn Tai Li Min of the School of Medical Sciences, Universiti Sains Malaysia, to the Editorial Board of MJMS. She has been appointed as a subeditor of MJMS, effective 1 May 2021.

In early July 2021, we also welcomed other new members to the statistical editor team of MJMS: Ms Fairuz Fadzilah Rahim of the Royal College of Surgeons in Ireland (RCSI) and University College Dublin (UCD) Malaysia Campus, Pulau Pinang, Malaysia; Mr Hazwan Mat Din of Universiti Putra Malaysia, Selangor, Malaysia; and Dr. Nurzulaikha Mahd Ab.lah of the School of Medical Sciences, Universiti Sains Malaysia, Kelantan, Malaysia.

## Figures and Tables

**Table 1:** Countries or regions that have contributed new manuscripts to MJMS in the most recent 3-year period (2019–2021)

Country/Region	Manuscript average count
Malaysia	182
Iran	27
Indonesia	21
India	16
England	9
Australia	8
Nigeria	7
Thailand	7
Saudi Arabia	6
Pakistan	5
Brunei	4
USA	4
Singapore	3
Egypt, Sudan, Taiwan and Turkey	2
Belgium, Brazil, Bulgaria, China, France, Germany, Ghana, Italy, Japan, Jordan, Kenya, Scotland, South Africa, South Korea, Tanzania, Vietnam and Yemen	1

**Table 2:** Accepted manuscripts by country/region with a decision date between 1 January 2021 and 31 December 2021

Country/Region	Total manuscript	Percentage of acceptance (%)
Malaysia	283	33.57
Indonesia	97	12.37
Iran	85	12.94
India	79	2.53
Brunei	5	60.00
Jordan	5	0.00

Source: https://mc.manuscriptcentral.com/maljms

**Table 3:** Major contributors by institution in the most recent 3-year period (2019–2021)

Organisation	Manuscript average count
Universiti Sains Malaysia	100
Universiti Kebangsaan Malaysia	26
Ministry of Health Malaysia	16
Universiti Malaya	13
Universiti Teknologi MARA	13
Universiti Putra Malaysia	12
University of Indonesia	10
International Islamic University Malaysia	9
Hospital Kuala Lumpur	8
Islamic Azad University	7
Sarawak General Hospital	7
Universiti Sultan Zainal Abidin	6
Kermanshah University of Medical Sciences, Shahrekord University of Medical Sciences, Tehran University of Medical Sciences and Universiti Malaysia Sabah	5
Gadjah Mada University, Hospital Sungai Buloh, Iran University of Medical Sciences and University Brunei Darussalam	4

**Table 4:** Impact scores and CiteScore trends

Year	Impact score	CiteScore
2021	1.82	2.5
2020	1.39	2.0
2019	0.91	1.4
